# DNA binding properties of human Cdc45 suggest a function as molecular wedge for DNA unwinding

**DOI:** 10.1093/nar/gkt1217

**Published:** 2013-11-28

**Authors:** Anna Szambowska, Ingrid Tessmer, Petri Kursula, Christian Usskilat, Piotr Prus, Helmut Pospiech, Frank Grosse

**Affiliations:** ^1^Research Group Biochemistry, Leibniz Institute for Age Research -Fritz Lipmann Institute, Beutenbergstrasse 11, D-07745 Jena, Germany, ^2^Laboratory of Molecular Biology IBB PAS, Affiliated with University of Gdansk, Wita Stwosza 59 Gdansk, Poland, ^3^Rudolf Virchow Center, DFG Research Center for Experimental Biomedicine, Josef Schneider Strasse 2, 7080 Wurzburg, Germany, ^4^Department of Biochemistry, Oulu, P.O. Box 3000, University of Oulu, Oulu 90014, Finland, ^5^Department of Chemistry, University of Hamburg/DESY, Notkestrasse 85, 22607 Hamburg, Germany, ^6^Biocenter Oulu, P.O. Box 3000, University of Oulu, Oulu 90014, Finland and ^7^Center for Molecular Biomedicine, Friedrich-Schiller University, Biochemistry Department, Jena, Germany

## Abstract

The cell division cycle protein 45 (Cdc45) represents an essential replication factor that, together with the Mcm2-7 complex and the four subunits of GINS, forms the replicative DNA helicase in eukaryotes. Recombinant human Cdc45 (hCdc45) was structurally characterized and its DNA-binding properties were determined. Synchrotron radiation circular dichroism spectroscopy, dynamic light scattering, small-angle X-ray scattering and atomic force microscopy revealed that hCdc45 exists as an alpha-helical monomer and possesses a structure similar to its bacterial homolog RecJ. hCdc45 bound long (113-mer or 80-mer) single-stranded DNA fragments with a higher affinity than shorter ones (34-mer). hCdc45 displayed a preference for 3′ protruding strands and bound tightly to single-strand/double-strand DNA junctions, such as those presented by Y-shaped DNA, bubbles and displacement loops, all of which appear transiently during the initiation of DNA replication. Collectively, our findings suggest that hCdc45 not only binds to but also slides on DNA with a 3′–5′ polarity and, thereby acts as a molecular ‘wedge’ to initiate DNA strand displacement.

## INTRODUCTION

Faithful duplication of eukaryotic DNA requires the ordered and highly coordinated recruitment of many proteins into the origins of replication to form a replication initiation complex (1–5). The human Cdc45 protein (hCdc45) is necessary and essential for both the establishment (6–8) and progression of the replication fork (9–11). Together with the four subunits of the GINS complex (go-ichi-nii-san, Japanese for 5-1-2-3, for the subunits Sld5, Psf1, Psf2 and Pfs3), Cdc45 associates with the heterohexameric Mcm2-7 helicase and forms the Cdc45-Mcm2-7-GINS (CMG) complex. CMG represents the eukaryotic replicative DNA helicase that unwinds origins of replication and drives the replication fork (12–14). Compared with Mcm2-7 alone, the CMG complex exhibits a much stronger DNA helicase and ATPase activity (15), indicating that Cdc45 and GINS are true subunits of the replicative DNA helicase. This was confirmed by single-particle electron microscopy studies showing that the Mcm2-7 complex forms an open lock-washer structure, and binding of Cdc45 and GINS causes a closed-ring formation (16). Apparently, this conformational change is mandatory to efficiently activate the helicase function of CMG (15).

Many reports have shown that Cdc45 also interacts with several other replication proteins, such as the replicative DNA polymerases alpha, delta and epsilon, MCM10, Orc2 and TopPB1 (17,18). Despite the crucial role of Cdc45 in eukaryotic replication, its biochemical functions are still enigmatic. Recent bioinformatics approaches have revealed sequence similarities between the N-terminal-conserved DHH domain of Cdc45 and bacterial RecJ (19–21). Members of the DHH phosphoesterase superfamily are defined by the presence of four sequence motifs containing conserved aspartic acid (D) and histidine (H) residues that are essential for Mn^2+^ or Mg^2+^ binding and catalysis (20). The aspartic acid–histidine-pair has been postulated to act as general acid that protonates the oxygen between two phosphate groups, thereby facilitating hydrolysis (22,23).

The structure and function of pyrophosphatases of family II that belong to the DHH superfamily have been extensively studied, and the results suggest a three-metal catalyzed mechanism for pyrophosphate hydrolysis with a preference for transition metals over Mg^2+^ for coordination (24). In addition, the biochemical properties of two other proteins of the DHH family have been characterized, namely, RecJ and the scPPX1 protein from *S. cerevisiae* (25,26). Bacterial RecJ is an Mn*^2+^*- or Mg^2+^-dependent exonuclease that degrades single-strand DNA (ssDNA) in the 5′–3′ direction in a highly processive manner (27). RecJ plays an important role in DNA replication, repair and recombination (27–31), whereas yeast scPPX1 degrades polyphosphates (32). The crystal structure of a truncated *Thermus thermophilus* RecJ protein reveals a typical DHH superfamily fold consisting of two globular domains interconnected by a long alpha-helix. A positively charged central groove for the binding of ssDNA and Mn^2+^ is located between the two domains (23,33).

Here, we present a structural characterization of hCdc45 and analysis of its DNA-binding properties. By using several biophysical methods, we found that hCdc45 is a folded protein that forms monomers in solution and displays a predominantly alpha-helical structure. *Ab initio* and rigid body shape reconstructions based on small-angle X-ray scattering (SAXS) further confirm that hCdc45 adopts a DHH core-like form similar to RecJ, and that it possesses an extension indicating the presence of a large insertion compared with its bacterial homolog. Our DNA-binding studies revealed that hCdc45 bound weakly to a 34-mer ssDNA and much strongly to an 80 or a 113-mer. hCdc45 displayed an even higher affinity to Y-form and bubble DNA structures, which are regarded as intermediates of DNA replication. Moreover, hCdc45 seems to slide along ssDNA with a 3′–5′ polarity until it reaches an ss/double-strand DNA (dsDNA) junction, consistent with a wedge function of Cdc45 within the CMG complex.

## MATERIALS AND METHODS

### Reagents

Agar, yeast extract and tryptone broth were obtained from Carl Roth KG (Karlsruhe, Germany). T4 polynucleotide kinase and TrueStart Hot Start Taq DNA polymerase were purchased from Thermo Scientific (Schwerte, Germany). Plasmid pUC18 and Nt. BspQI (nickase) were obtained from New England Biolabs. All other chemicals were from Sigma-Aldrich (Germany).

### Oligonucleotides

Oligonucleotides were purchased in triple high-performance liquid chromatography-purified quality from Purimex (Grebenstein, Germany), and their sequences are described in the Supplementary Table S2. The concentrations were determined spectrometrically. Supplementary Table S1 contains oligonucleotides used to assemble the individual DNA substrates in this study.

### Cloning, expression and purification of C-terminally His_6_-tagged hCdc45

The full-length open reading frame of hCdc45 was amplified from human genomic DNA by polymerase chain reaction (PCR) using appropriate primers (Supplementary Table S2). The amplified fragment was subcloned into the pRSFDuet-1 vector (Novagen) using the restriction sites PciI and XhoI. Expression of hCdc45-His_6_ was carried out in the *Escherichia coli* strain BL21 Rosetta (DE3) by overnight incubation at 16°C, and induction with 0.3 mM IPTG. After centrifugation, the bacterial cell pellet was resuspended in the lysis buffer consisting of 50 mM NaH_2_PO_4_ pH 7.6, 300 mM NaCl, 1 mM MgCl_2_, 15 mM imidazole, 2 mM β-mercaptoethanol, 0.05% Triton X-100, 5% glycerol and 2 ml/g of bacterial pellet of the ethylenediaminetetraacetic acid (EDTA)-free protease inhibitor cocktail from Roche (Basel, Switzerland). After incubation on ice for 15 min, 1-mg lysozyme per milliliter bacterial suspension was added, followed by 35-min incubation on ice. Bacterial cells were disrupted by sonication, and insoluble material was removed by centrifugation at 35 000 × *g* for 45 min at 4°C. The clear supernatant was transferred to a test tube and incubated with 4-ml Ni-NTA agarose (Qiagen, Hilden Germany) under gentle rotation for 1.5 h at 4°C. Then, the resin was packed into a column and washed with 30 ml of lysis buffer, followed by 15 ml of 25 mM imidazole (wash-1 buffer) and 15 ml of 40 mM imidazole (wash-2 buffer). hCdc45 was eluted with 15 ml of 100 mM imidazole (elution-1 buffer), followed by 15 ml of 150 mM imidazole (elution-2 buffer). After pooling the Cdc45-containing fractions, the buffer was exchanged with an Amicon concentrator 30 k (Millipore). The concentrated fractions were loaded onto a 0.5 × 3-cm Q-Sepharose fast flow column (GE Healthcare) equilibrated with buffer A (50 mM HEPES-KOH, pH 7.5, 150 mM KCl, 1 mM DTT, 1 mM MgCl_2_ and 10% glycerol). The column was washed with 15 ml of buffer A and then with 10 ml of buffer A plus 250 mM KCl. Cdc45 was eluted from the column with 15 ml of buffer B (50 mM HEPES-KOH, pH 7.5, 400 mM KCl, 1 mM DTT, 1 mM MgCl_2_ and 10% glycerol). Fractions containing hCdc45 were pooled and concentrated with an Amicon concentrator as above and loaded onto a Superose 6 gel filtration column (GE Healthcare) equilibrated with buffer C (20 mM HEPES-KOH, pH 7.5, 150 mM KCl, 2 mM DTT and 5% glycerol). The eluted protein was collected and the purity was analyzed on a 10% sodium dodecyl sulphate-polyacrylamide gel electrophoresis (SDS-PAGE). The protein concentration was determined using the protein-assay kit from Bio-Rad.

### Radioactive labeling and preparation of DNA substrates

The oligodeoxyribonucleotides used for the preparation of DNA substrates [for electrophoretic mobility shift assay (EMSA) and surface plasmon resonance (SPR) experiments] are listed in the Supplementary Table S2. Before annealing, the 5′-ends were labeled with 33PγATP (5000 Ci/mmol) from Hartmann Analytic (Braunschweig, Germany) and T4 polynucleotide kinase for 1 h at 37°C in forward reaction buffer (80 mM Tris–HCl, pH 7.4, 10 mM MgCl_2_ and 5 mM dithiothreitol). The reaction was stopped by heating to 72°C for 10 min. Subsequently, unincorporated nucleotides were removed by size-exclusion chromatography using Ilustra MicroSpin G-25 columns (GE Healthcare). To prepare different DNA substrates, the 5′-end-labeled oligonucleotides were mixed with unlabeled DNA (at a molar ratio of 1:1.2) in binding buffer (30 mM Tris–HCl, pH 7.5, 10 mM MgCl_2_), heated to 95°C for 5 min and left to cool down to room temperature.

### EMSAs

DNA binding reactions (10 µl) were prepared in 50 mM HEPES-KOH, pH 7.5, 0.5 mM DTT, 150 mM KCl and 50 µg/ml bovine serum albumin. Each reaction mixture contained either 2 or 4 nM 5′-end-labeled DNA and the indicated amounts of Cdc45. The solutions were incubated for 10 min at 30°C and then rapidly cooled on ice. Then, 5 µl of loading buffer (0.1% bromophenol blue, 0.1% xylene cyanol and 40% glycerol) was added. Next, the samples were separated on 10% native polyacrylamide gels in 89 mM Tris base, 89 mM boric acid and 2 mM EDTA (1 × TBE) buffer. After electrophoresis, the gels were dried for 1 h at 80°C, exposed to a phosphorimaging screen and visualized with a Phosphor-Imager (Typhoon Trio, GE Healthcare, Germany).

### Preparation of DNA substrates for atomic force microscopy

Fragments of 270 bp and 548 bp of pUC18 DNA (spanning residues 453–723 and 643–1191, respectively) were obtained by PCR amplification using the primer pair pUC18-453F and pUC18-723R, and pUC18-643F and pUC18-643R (Supplementary Table S2), and relaxed-circular pUC18 as template. Subsequently, the linear fragments were purified by agarose gel electrophoresis and recovered using the PCR clean-up kit from Qiagen (Hilden, Germany). To prepare DNA substrates with 40-nt-long 3′ and 5′-overhangs, the DNA fragments were annealed with a 10-fold excess of complementary oligonucleotide to deplete the short ssDNA stretch, between the DNA fragment end and the nick site, for 5 min at 95°C. The mixture was immediately spin-filtered through a 50-kDa MWCO filter, corresponding to a 125-nt ssDNA cutoff (Millipore). Filtration was repeated at least twice. Finally, the DNA substrates were purified by agarose gel electrophoresis and recovered using the PCR clean-up kit from Qiagen.

### Atomic force microscopy

For atomic force microscopy (AFM) imaging of Cdc45, the protein was diluted to 10 nM in AFM deposition buffer *(*20 mM HEPES, pH 7.5, 25 mM sodium-acetate, 10 mM magnesium-acetate), and a small volume (20 µl) of the protein solution was deposited onto freshly cleaved mica, rinsed with ultrapure deionized water and dried in a stream of nitrogen gas. For imaging of Cdc45 binding to DNA substrates, the DNA (see above) was pre-incubated at 65°C for 10 min and slowly cooled down to room temperature to remove any salt crystals formed during storage. Protein (0.75 μM) and DNA (90–100 nM) were incubated in buffer containing 20 mM Tris HCl, pH 7.5, 150 mM NaCl, 10 mM DTT and 10 mM CaCl_2_ for 15 min at ambient temperature. Samples were then diluted 40–50-fold in AFM deposition buffer (see above) and deposited onto freshly cleaved mica, as described above. AFM scans were performed on an MFP 3D BIO atomic force microscope (Asylum Research) in oscillating mode using Olympus OMCL AC240 silicon probes with spring constants of ∼2 N/m and resonance frequencies of ∼70 kHz. Images were captured at scan speeds of 2.5 µm/s and at scan sizes of 1 × 1, 2 × 2 or 4 × 4 μm^2^ with corresponding pixel resolutions of 512 × 512, 1024 × 1024 and 2048 × 2048, respectively. For analysis, AFM images were flattened to third order. AFM volumes (of free protein molecules as well as DNA-bound complexes) were measured using ImageSXM (S. Barret) software and translated into protein molecular weights via a standard linear relationship [V = 1.2 × (MW) − 5.9, where V is the AFM volume and MW is protein molecular weight]. Molecular weights were derived from the center position of a Gaussian fit to the distribution of measured volumes [±1 standard deviation (SD)] using the software Origin. Analyses of protein binding to different DNA end structures (blunt and 40-nt 3′ or 5′-overhangs) include only protein peak volumes bound to DNA fragment ends. Maximum diameters of Cdc45 molecules (Dmax) were determined as the center of Gaussian fits to statistical distributions of AFM tip contribution corrected lengths of the major axes of elliptical fits to the particles in the images. Similarly, *R*g values were determined as the average (tip contribution corrected) half length of minor and major axes from the elliptical fits to the particles. Correction for AFM tip convolution effects was achieved as previously described (34).

### Real-time analysis of Cdc45-DNA interaction by SPR

The interactions between Cdc45 and various DNA substrates were measured with a BIAcore T200 optical biosensor (GE Healthcare) at 25°C. First, the His6-tagged Cdc45 protein was immobilized on the surface of the CM5 chip from BIAcore using the His-capture kit (GE Healthcare) by following the instructions of the manufacturer. The anti-His antibody was immobilized to yield an optical signal of ∼1200 RU. Residual activated groups were blocked by a 7-min injection of 1 M ethanolamine (pH 8.5) according to the BIAcore immobilization procedure. Experiments were carried out using 5-min injections of different concentrations of DNA substrates in HBS buffer (20 mM HEPES-KOH, pH 7.5, 150 mM KCl, 1 mM DTT, 3 mM EDTA and 0.005% surfactant P20). DNA substrates were prepared as described above. The HBS buffer was injected over the chip, and the dissociation phase was recorded. The analyses were performed at a flow rate of 30 µl/min. Following completion of DNA injection, the surface of the chip was regenerated by applying 0.5 M NaCl. Data were evaluated using the Biacore T200 evaluation software v1.0, after subtraction of the background response signal and correction of the buffer effect. As a control for non-specific interactions, an empty reference cell was used. Dissociation constants were calculated from the kinetic rate constants for DNA/Cdc45 complex formation, and dissociation was derived from a 1:1 interaction model and from a concentration-dependent steady-state binding of various DNA molecules.

### Tryptophan fluorescence spectroscopy

Fluorescence was recorded by a Carry Eclipse fluorescence spectrophotometer (PerkinElmer Ltd., England) using FL WinLab software. Thirty micrograms of hCdc45 in 40 mM HEPES-KOH, pH 7.5, 200 mM KCl, 1 mM MgCl_2_, 1 mM DTT and 20% glycerol was centrifuged at 10 000 rpm for 40 min before the measurements. All experiments were carried out in a 1 × 1-cm quartz cuvette at an excitation wavelength of 295 nm. Fluorescence emission was measured at different wavelengths after adding increasing concentrations of guanidinium hydrochloride. After each measurement, 25-µl aliquots were removed from the cuvette and replaced by the same volume of 8 M guanidinium hydrochloride. The solution was mixed and incubated for 10 min at room temperature and then measured. Titrations were repeated several times until the protein was completely denatured.

### Synchrotron radiation CD spectroscopy

Synchrotron radiation circular dichroism (SRCD) spectra were recorded at the ultraviolet (UV)-CD12 beamline of the ANKA storage ring in Karlsruhe, Germany. Before measurements, hCdc45 was dialyzed against 10 mM potassium phosphate (pH 7.5) containing 150 mM NaF. For comparison, SRCD spectra were also measured in storage buffer (20 mM HEPES-KOH, pH 7.6, 150 mM KCl, 2 mM DTT, 2 mM MgCl_2_ and 5% glycerol), which, however, had a high UV absorption. SRCD spectra were collected at 4.7 mg/ml in the wavelength range of 170–280 nm using a 13-µm path-length CaF_2_ cuvette. Three consecutive scans were recorded and averaged, and the signal of an identically measured buffer was subtracted. Data processing was carried out using the CDtool Software (35–37). Secondary structure deconvolution was carried out at the Dichroweb server (38), with the CDSSTR algorithm and the SP175 reference data set (39). The use of other deconvolution algorithms (SELCON, CONTINLL, VARSLC and K2d) or reference data sets produced comparable estimates of the secondary structure. Normalized root-mean-square deviations indicated the most accurate fit for the spectra.

### Small-angle X-ray scattering

Synchrotron SAXS data were collected on the beamline I911-4 at MAX-Lab, Lund (Sweden). X-ray scattering patterns were recorded with protein concentrations between 1.3 and 5.4 mg/ml in 20 mM HEPES-KOH, pH 7.5, 150 mM KCl, 1 mM DTT, 1 mM MgCl_2_ and 5% glycerol. The data from three different measured concentrations were evaluated for analysis by using programs of the ATSAS suite (40). PRIMUS (41) was used for SAXS data processing and analysis, including radius of gyration (*R*_g_) determination with the Guinier method. The *R*g value and maximum distance (D_max_) were evaluated using GNOM (42), which was also used to calculate the distance distribution functions. *Ab initio* models of hCdc45 were built using GASBOR (43) and DAMMIF (44). In the latter case, a number of individual structures were averaged using SUPCOMB (45) and DAMAVER (46). While DAMMIF prepares a model of dummy atoms within a 3D envelope, GASBOR builds a protein-like chain of the correct length from dummy residues. Comparisons with the crystal structure of *T. thermophilus* RecJ (PDB 1IR6) (23) were carried out using CRYSOL (47). Based on the RecJ structure, secondary structure predictions and the sequence alignment, rigid body refinement coupled to loop building was also performed with the program BUNCH (48). In BUNCH modeling, models of the N- and C-terminal domains were used as two rigid bodies, and the central part (residues 141–420 of hCdc45), which corresponds to an insertion in hCdc45 compared with RecJ, was built *ab initio*. Possible conformational ensembles were studied with the same approach using EOM (49), but only poor fits to the raw data were obtained. Additional analyses of the SAXS data were carried out with the scÅtter program (https://bl1231.als.lbl.gov/scatter/), including molecular weight estimation based on V_c_ (50). In the latter case, *Q_R_* was defined as the ratio between V_c_^2^ and R_g_, and the molecular weight, in turn, was obtained as MW = Q_R_/0.1231. The Porod volume *V_p_* was estimated using DATPOROD (40), and the Porod invariant *Q* was further calculated using the relationship Q = 2π^2^ I(0)/V_p_.

### Limited proteolysis

Limited proteolysis was performed using elastase, V8 proteinase and trypsin. Briefly, 50 µg of Cdc45 were incubated in 100 µl of 20 mM Tris-HCl, pH 7.5, and 150 mM NaCl at 25°C with 1 µl of 0.5 mg/ml elastase and V8 proteinase and 0.05 mg/ml trypsin, respectively. At different time points, 10-µl aliquots of the reaction mixture was withdrawn, and the reaction was stopped by the addition of 1 µl of inhibitor (100 mM phenylmethanesulfonyl fluoride) and 3 µl of 4 × SDS-PAGE loading dye. The reaction products were analyzed by SDS-PAGE. Bands were characterized by Edman sequencing using a PRocise 494A device from Applied Biosystems.

### *In silico* analysis

Regions of disorder were identified with I-Poodle (51).

## RESULTS

### Purified Cdc45 is a folded monomeric protein

**Figure 1.**
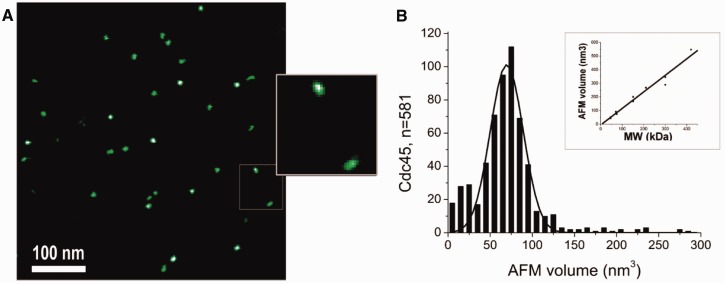
hCdc45 represents a well-folded monomer in solution. (**A**) AFM imaging of hCdc45 reveals a homogeneous population of monomeric protein molecules in solution. The scale bar corresponds to 100 nm. The enlarged inset shows examples of elongated Cdc45 shapes. (**B**) AFM volume analysis indicates a molecular mass of 63 kDa for human Cdc45, consistent with a homogeneous population of monomeric protein molecules in solution. AFM volumes are translated into protein molecular weight based on a linear calibration curve (inset).

Human Cdc45 was expressed in and purified to homogeneity from bacterial cells as described in Materials and Methods section (Supplementary Figure S1). The quality of the purified protein was controlled by dynamic light scattering, SAXS and atomic force microscopy (AFM), all of which indicated a monodisperse behavior. The molecular mass was determined as 75 kDa using the intensity of SAXS forward scattering compared with that of bovine serum albumin, and 63 ± 19 kDa using AFM (Figure 1). In addition, the molecular weight was determined using a concentration-independent method, as recently described (Supplementary Figure S2D) (50). The V_c_ parameter determined from the SAXS data was 603 Å^2^, corresponding to a molecular weight of 85 kDa (50). The Porod volume of 144 nm^3^ further indicates a MW of 70–90 kDa (52), suggesting monomeric state, taking into account the relatively inflated volume/MW ratio of non-globular particles. The Porod invariant based on this analysis was 0.013 Å^−^^3^.These data are in agreement with analytical gel filtration (Supplementary Figure S1) and in line with the calculated monomeric molecular mass of 66 kDa. Tryptophan fluorescence spectroscopy revealed a largely folded protein that required nearly 3 M guanidinium hydrochloride for full denaturation (Supplementary Figure S1E and F).

### Structure of Cdc45

**Figure 2.**
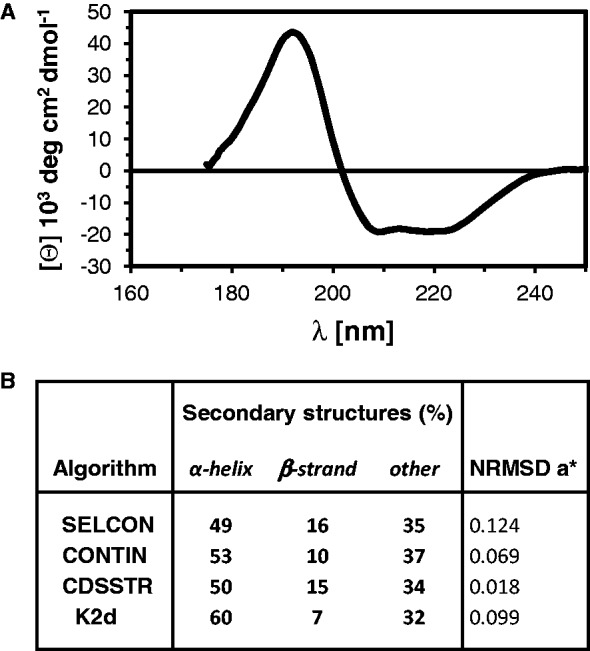
Synchrotron radiation circular dichroism spectrum of hCdc45. (**A**) The spectrum was recorded at the UV-CD12 beamline of the ANKA storage ring, Karlsruhe, Germany, in 10 mM potassium phosphate, pH 7.5, and 150 mM NaF. The ellipticities θ, in deg dmol-cm, is plotted as a function of wavelength. Data processing was carried out using the CDtool software. (**B**) The secondary structure elements of hCdc45 were assessed using the indicated algorithms and the SP175 reference dataset, available on the DichroWeb server. An asterisk indicates the normalized root-mean-square deviation between calculated and experimental spectra.

To obtain information on secondary structure elements of hCdc45, we performed SRCD measurements. SRCD is advantageous over traditional circular dichroism because it enables collection of data at lower wavelengths and thus provides more detailed structural information. The collected spectra displayed two negative maxima of ellipticity at 208 and 220 nm, and a strong maximum at 193 nm, that are typical for mostly alpha-helical proteins (Figure 2). Deconvolution of these data yielded an estimated secondary structure composition of 50% alpha-helix, 15% beta-strand, 10% turns and 24% unordered structure (Figure 2). The secondary structure content was close to that predicted from the sequence and consistent with the proposed homology to members of the DHH family of phosphoesterases. As observed by others (20–22), our bioinformatics analyses also confirmed that hCdc45 is a distant RecJ/DHH family member.

**Figure 3.**
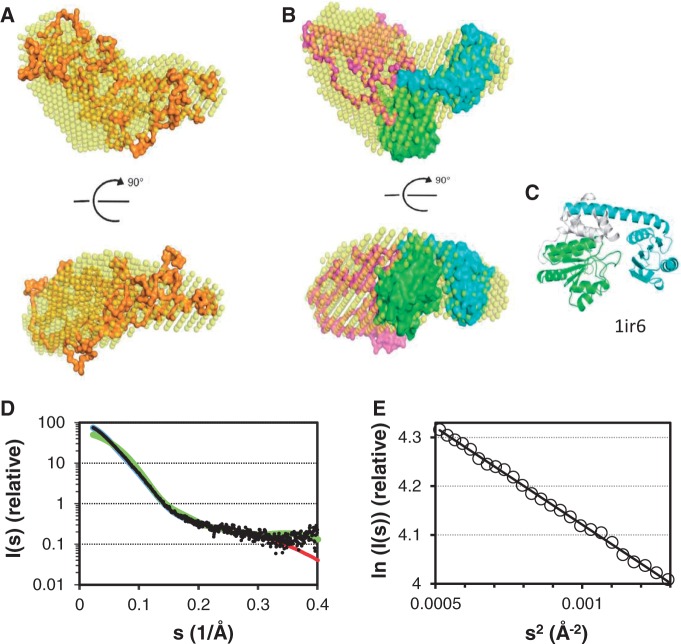
Three-dimensional structural models of hCdc45 protein. (**A**) Superposition of the GASBOR and DAMMIF models. (**B**) Superposition of the DAMMIF and BUNCH models. The *ab initio* bead model of hCdc45 in (A) and (B) is presented as transparent yellow spheres, the model from GASBOR as an orange surface, the model from BUNCH as a molecular surface with the N-terminal domain in green, the C-terminal domain in turquoise and the helical insertion or loop in magenta. The two figures are related by a rotation of 90° around the abscissa. (**C**) The crystal structure of *T. thermophilus* RecJ (1IR6) with the same coloring as in the BUNCH model for comparison. The BUNCH model is based on the N-and C-terminal domains of RecJ, whereas the gray part, corresponding to the linker and insertion, was rebuilt in the modeling. (**D**) SAXS curve of hCdc45. The experimental SAXS profile of human Cdc45 protein (black dots) is compared with the theoretical scattering curves calculated from the BUNCH model (red), the DAMMIF model (blue) and the RecJ crystal structure (1IR6) (green). (**E**) Guinier plot of hCdc45. The linearity of the Guiner plot (plotted in the range of 0.8 < sRg < 1.3) indicates the quality of the experimental SAXS data.

The DHH core, exemplified by the crystal structure of *T. thermophilus* RecJ (pdb 1ir6) (23), displays two non-similar globular domains connected by a long alpha-helix. To obtain more structural information on hCdc45 in solution, we performed SAXS experiments (Figure 3). Based on the distance distribution function, hCdc45 has a maximum dimension of ∼12 nm (Supplementary Figure S2), and based on the Guinier plot (Figure 3D), *R*_g_ of 3.6 nm, consistent with the dimensions observed by AFM (Dmax, AFM = (12.2 ± 3.2.) nm, (R_g_),AFM = (4.3 ± 0.7) nm (Supplementary Figure S6). *Ab initio* shape reconstructions are in agreement with a DHH core-like structure similar to RecJ, with an additional extension, indicating a large insertion in Cdc45 compared with RecJ (Figure 3A and B). This insertion may include a disordered region—between amino acids 113 and 185—that was predicted by bioinformatics analysis and experimentally confirmed by limited proteolysis (Supplementary Figure S3). On the other hand, an analysis of the SAXS data by way of plotting the dimensionless Kratky plot (53,54) indicates very little flexibility of hCdc45 (Supplementary Figure S2E). More specifically, for a rigid globular particle, a maximum (with a value of 1.104) in this plot is expected at s*R_g_ = √3 ≈ 1.73 (52). For hCdc45, the strong maximum (value 1.2) is located at s*R_g_ = 2.0, which is very close to the expected value for an ordered globular particle. An increasing content of unfolded structure would move the maximum toward larger s*Rg values, eventually resulting in a plateau for a fully disordered molecule (53,54), and there is no indication of this in the hCdc45 SAXS data.

For a detailed insight into the structure of hCdc45 in solution, molecular modeling was carried out based on the SAXS data. In addition to bead-based and chain-like models, a mixed approach was uptaken, whereby models of the N-and C-terminal domains were used as rigid bodies, and the insertion (compared with RecJ) was modeled *ab initio*. Assuming that the N- and C-terminal domains (shown in green and turquoise, respectively, in Figure 3B) are in proximity to each other, and the long helix is positioned before the C-terminal domain, the models suggest that the additional domain (cyan, Figure 3B) is in contact with the N-terminal domain of the DHH core of hCdc45. Models built by different methods (Figure 3 and Supplementary Figure S2) were all highly similar in shape, showed excellent fits to the experimental data (Figure 3C and Supplementary Figure S2B) and were compatible with a structure containing the expected DHH core, with an additional extension.

### Length-dependent binding of Cdc45 to ssDNA

**Figure 4.**
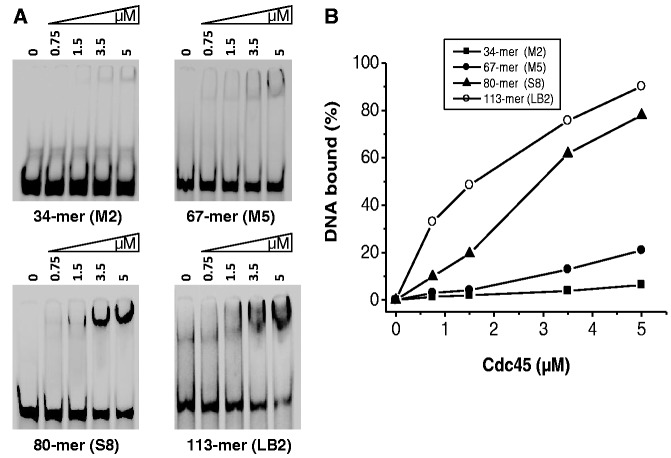
Binding of hCdc45 to ssDNA substrates. (**A**) Electrophoretic mobility shift assays were performed with 2 nM 5′-labeled ssDNA and the indicated amounts of hCdc45. After 10 min at 30°C, the reaction mixtures were cooled to 0°C. After addition of 5-µl loading dye, the samples were electrophoresed through a 10% non-denaturing polyacrylamide gels in 1× TBE buffer. Subsequently, gels were dried and exposed to a phosphorimaging screen. Gels were visualized using a phosphor-imager (Typhoon Trio; GE Healthcare). (**B**) The graph represents the percentage of bound DNA per added concentration of hCdc45 as calculated by the Image Quant software.

The structural similarity to RecJ and the fact that hCdc45 is a component of the replication fork both suggest that Cdc45 binds DNA. To address this question experimentally, we performed detailed DNA binding analyses using electrophoretic mobility shift assays (EMSAs), SPR and AFM. First, we analyzed hCdc45 binding to different ssDNA substrates (Supplementary Table S1). EMSAs were performed by incubating increasing amounts of hCdc45 with 2-nM DNA for 10 min at 30°C. As its homolog from yeast, hCdc45 bound a 113-mer ssDNA much better than a 67-mer, whereas a rather weak binding was observed for a 34-mer (Figure 4). Although the overall affinity of hCdc45 to ssDNA was weak (5 µM Cdc45 retained only 85% of 2 nM of the 113-mer), binding to the 113-mer ssDNA was ∼8.5-fold more efficient than binding to the 67-mer (Figure 4B). Better binding to longer single strands is surprising, as from the RecJ-like structure a binding site size of only 7 nt would be expected.

### Cdc45 binds branched DNA structures tighter than to ssDNA

**Figure 5.**
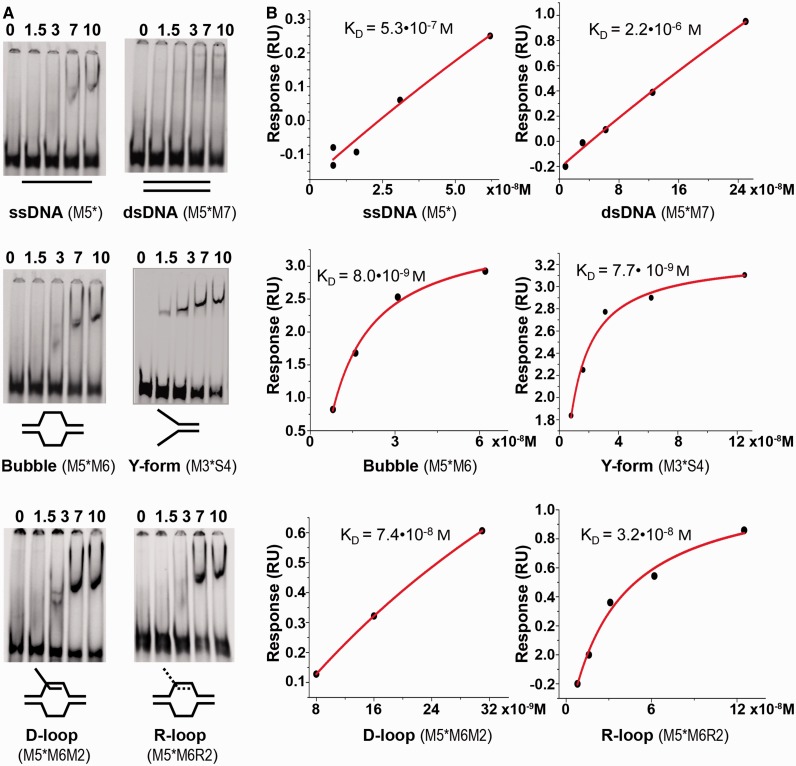
Binding of hCdc45 to different DNA structures. (**A**) Electrophoretic mobility shift assays were performed with 4 nM 5′-labeled DNA substrates and the indicated amounts of hCdc45. Gel electrophoresis was as described in the legend for Figure 4. The used oligonucleotides are listed in parentheses. The asterisks (*) represent the 5′-label. (**B**) Real-time binding analysis of various DNA substrates to hCdc45. Direct interaction between Cdc45 and DNA substrates was monitored using the BIAcore system. Binding was measured at 25°C at a flow rate of 30 µl/min in 20 mM HEPES-KOH, pH 7.5, 150 mM KCl, 1 mM DTT, 3 mM EDTA and the surfactant P20. The dissociation constants were calculated from the concentration-dependent steady-state response of DNA binding.

**Figure 6.**
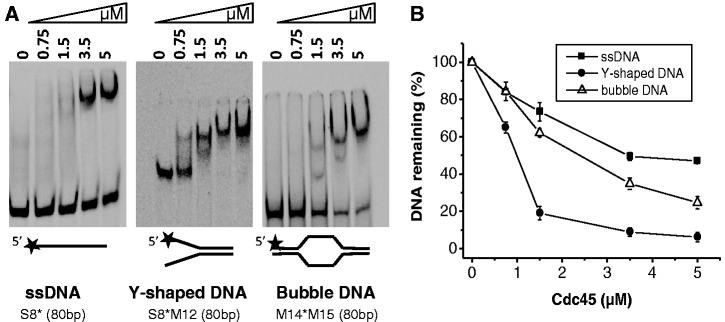
hCdc45 binding to branched DNA structures. (**A**) Electrophoretic mobility shift assays were performed using 2 nM 5′-labeled DNA substrates and the indicated amounts of hCdc45 as described in the legend for Figure 4. (**B**) The graph represents the percentage of unbound DNA as function of the hCdc45 concentration. The mean standard error from three independent experiments is also given. Densitometric analyses were performed using the Image-Quant software.

Next, we addressed whether hCdc45 binds DNA structures that transiently emerge during DNA replication. hCdc45 bound Y-shaped DNA consisting of 17-bp dsDNA and two 17-mer overhangs (M3S4, Supplementary Table S1) much better than the corresponding 34-mer ss- and dsDNAs, and even better than the 67-mer ssDNA or dsDNA, as seen from EMSA and SPR analyses (Figure 5). Sequence context had little influence on the Cdc45 binding to forked structures, whereas length effects were more pronounced; for instance, a Y-structure with 40-mer tails became shifted almost completely at 3.5-µM Cdc45 (Supplementary Figure S4). The migration data showed a slight increase at higher Cdc45 concentrations indicating that under these conditions, more than one molecule of Cdc45 was bound per Y-DNA (Figure 5, Supplementary Figure S4). Comparable binding was also observed for other branched DNA substrates (Supplementary Figure S4). Next, we constructed bubble and D-loop structures, consisting of 22-bp dsDNA, a 20-nt-long ssDNA region forming the bubble, followed by another 22-bp dsDNA (M5M6). For the D-loop structure M5M6M2, the 17-mer 5′-half of the 34-mer M2 was hybridized onto the M6-part of the bubble (Supplementary Table S1). EMSA experiments revealed relatively high affinity of hCdc45 for both substrates (Figure 5A), which was confirmed by SPR analyses of DNA binding to immobilized hCdc45. For Y-shaped and bubble DNA, K_D_ values of 7.7 nM and 8.0 nM, respectively, were determined by SPR, whereas D- and R-loops bound by at least an order of magnitude weaker (Figure 5B). These data indicate that DNA binding of Cdc45 is substantially enhanced by the presence of an ss/ds junction. The observed tighter binding of hCdc45 to longer ssDNA prompted us to test whether this trend was also true for longer Y-forms and bubbles. We constructed a Y-shaped DNA with 40-bp dsDNA on one end and two 40-nt-long single-strand overhangs. Again, hCdc45 bound the longer Y-molecules much tighter than the shorter one (Supplementary Figure S5). For instance, binding of hCdc45 to this 80-mer Y-shaped DNA was 3- to 5-fold tighter than that to the 34-mer Y-DNA (Figure 6A). Binding of hCdc45 to the long ss-, Y-shaped and bubble-forming DNAs revealed affinities with an order of Y-forms > bubbles >> single strands >> double strands.

### hCdc45 displays a 3′–5′ preference on branched DNA

**Figure 7.**
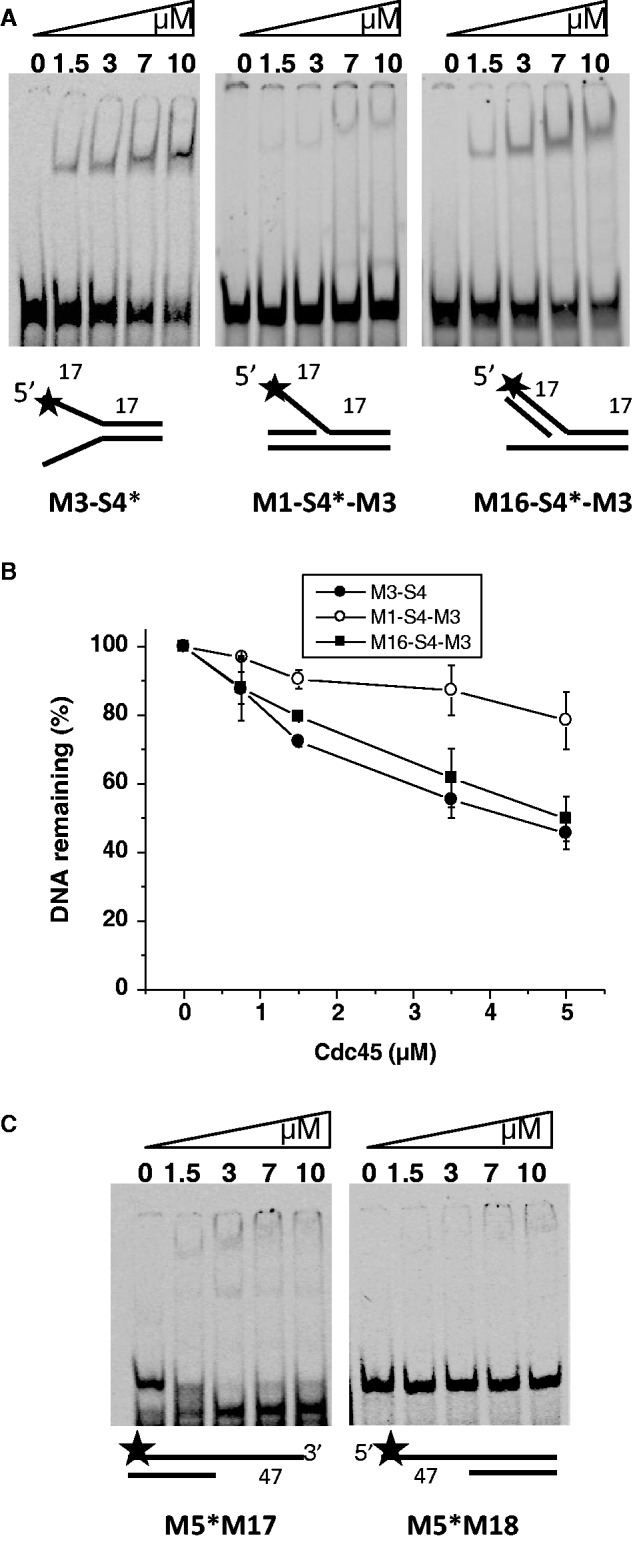
hCdc45 binds tightly to the 3′ arm of branched DNA structures. (**A**) hCdc45 binding to Y-shaped DNA and FLAP-DNA. Electrophoretic mobility shift assays were performed with 2-nM 5′-labeled DNA as described in the legend for Figure 4. (**B**) The graph represents the percentage of unbound DNA as function of the hCdc45 concentration with mean standard errors as described in the legend for Figure 6. (**C**) Binding of hCdc45 to 3′ and 5′-overhangs.

**Figure 8.**
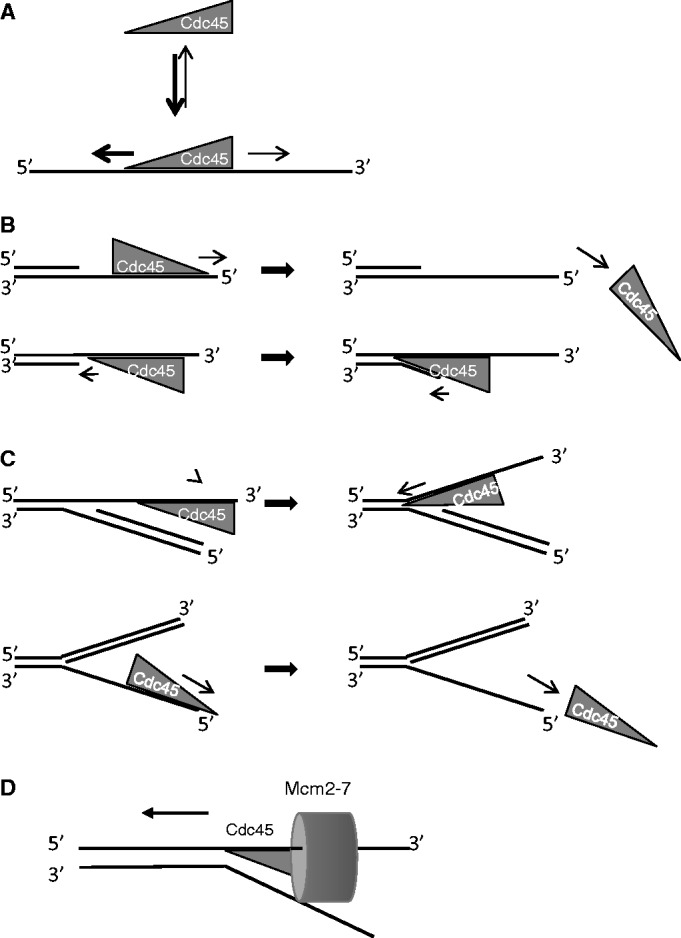
Hypothetical models for the action of hCdc45 on branched DNAs. (**A**) Binding of hCdc45 to ssDNA and sliding to the 5′ or 3′-end. (**B**) Preferred movement along ssDNA in the 3′–5′ direction aids to find the ss/ds junction (**C**) hCdc45 possesses a higher affinity for 5′-blocked flaps as compared with 3′-blocked structures. (**D**) Cdc45 may migrate in front of the Mcm2-7 helicase from 3′ to 5′ and, acting as a molecular ‘wedge’, to help displace the lagging strand.

The better binding to fork versus bubble structures indicated that hCdc45 might need a free end for attaching to DNA. To address this experimentally and to find out whether hCdc45 displays some binding polarity, we created Y-structures where either the 3′- or the 5′-overhang was blocked by hybridization with the complementary strand. Unexpectedly, the 3′-blocked Y-structure bound very poorly, whereas the 5′-blocked molecule bound nearly as well as the unblocked Y-form (Figure 7A and B), indicating a 3′–5′ preference of Cdc45 binding to the free single strand of the flap structure. This polarity of binding was not absolute, as there was residual binding of hCdc45 to the free 5′-end (Figure 7A and B). Comparable results were obtained by using dsDNAs with 3′- and 5′-overhangs. Although the amount of shifted DNA was low for both substrates, the 3′-overhanging DNA was shifted considerably better than DNA with a 5′-overhang (Figure 7C). This finding was corroborated by AFM analyses (Supplementary Figure S5), which demonstrated enhanced volumes of hCdc45 bound to DNA-ends with a 3′-overhang compared with a 5′-overhang or a blunt end. Overall, the data confirm a 3′-preference of binding in addition to the predominant binding to dsDNA junctions and ends. EMSAs also indicated some unwinding of a double-strand when Cdc45 was loaded onto substrate with free 3′-overhangs (Figure 7C), but not with 5′-overhangs. This sort of unwinding was passive, as it was detected in the absence of ATP and divalent cations. Collectively, these results may be explained by a model in which Cdc45 binds to ssDNA and either is able to slide along the ssDNA preferentially in 3′–5′ direction or multiple Cdc45 monomers successively loaded at the 3′-end work together to achieve a destabilization of the dsDNA helix, or a combination of both (Figure 8).

## DISCUSSION

Cdc45 represents an essential component of the eukaryotic DNA replication fork that is conserved from yeast to man. The importance of Cdc45, both initiation and propagation of DNA replication, prompted us to elucidate its structure and its molecular function in more detail. The highly purified recombinant protein was analyzed by several biophysical techniques. Analyses of the tryptophan fluorescence and SRCD spectra, together with AFM and DLS data, revealed that hCdc45 uniformly forms a highly ordered, mostly alpha-helical monomer in solution. An SRCD spectroscopy-based secondary structure calculation is in line with bioinformatics predictions proposing homology to the DHH superfamily. Low-resolution structural models of hCdc45, based on SAXS data, revealed an elongated molecule with a maximal dimension of 12 nm and *R*_g_ of 3.6 nm, as also supported by AFM analyses. A similar shape was earlier obtained using SAXS at a lower protein concentration, and an envelope-structure was built (20). The models are in agreement with a folded core with the approximate size of RecJ from the DHH family and an additional extension representing a large insert in hCdc45, compared with canonical DHH superfamily members (Figure 3A). Our findings are also consistent with the proposed structure of the CMG complex, obtained from single particle electron microscopy studies on *Drosophila melanogaster.* In this model, one molecule of Cdc45 and four subunits of GINS contribute to the formation of a closed-ring structure of the Mcm2-7 complex (15).

The evolutionary relationship of hCdc45 protein with DHH phosphoesterases, and, in particular, with bacterial RecJ, suggested a 5′–3′ exonuclease or a pyrophosphatase activity. However, neither activity could be detected by us or others (20). Even though hCdc45 retains a similar structure as its bacterial counterparts, it apparently lost its nuclease activity during evolution. Like bacterial RecJ, hCdc45 is able to bind ssDNA. Although the affinity of hCdc45 for ssDNA is weaker than that detected for the bacterial RecJ protein, we nevertheless could show that binding of hCdc45 to a 113-mer oligonucleotide was 8-fold stronger than binding to a 34-mer ssDNA (Figure 4). This largely confirms a study on Cdc45 from *S. cerevisiae*, where the authors postulated that binding of Cdc45 to long stretches of ssDNA (>60 nt) disrupts the CMG complex and thereby stalls the helicase (55). Based on the *R*_g_ of 3.6 nm, determined here, one molecule of Cdc45 may cover not >10–11 nt, while a binding site size of 7 nt has been determined for RecJ. These smaller binding site sizes hence do not explain a tighter binding of an 80-mer compared with a 34-mer. However, one explanation for this discrepancy may be that Cdc45 slides along ssDNA until it slips off the end. Thereby, it resides longer on a longer DNA as compared with a shorter one. In this respect, it is worth mentioning that the presumptive predecessors of hCdc45, RecJ and PPX1 are highly processive enzymes that degrade up to 1000 nt or polyphosphates per binding event. In RecJ, tight binding is achieved by forming a hole through which the ssDNA is threaded. The SAXS data do not support hole-formation of Cdc45, and dimerization has not been observed. However, the electron microscopy studies of the CMG complex suggest that Cdc45, together with GINS, may form such a hole distant from the central hole of the Mcm2-7 helicase. If such a hole is really taking up the displaced single strand, hCdc45 should bind ssDNA with a 5′–3′ polarity. However, our binding data to partially blocked Y-shaped DNAs and single-strand overhangs clearly suggest the opposite orientation, with tighter binding to free 3′ than 5′-overhangs, independent of whether they represent a simple ss/ds junction or are part of a Y-structure (Figure 7A and B). This polarity is hence the opposite of that observed for the bacterial homolog RecJ, that degrades ssDNA from the 5′-end. Nevertheless, we cannot exclude that *in vivo* Cdc45 might also act in the 5′–3′ direction, although the observed passive unwinding of double strands, on loading of Cdc45 on a 3′-overhang but not on a 5′-overhang, argues against such 5′–3′ directionality. In contrast, by moving in the 3′–5′ direction, hCdc45 is able to translocate to ss/ds junctions where it is then retained (Figure 8B). It may thus be involved in displacing the 5′-end of the complementary strand moving as a ploughshare or ratchet into a thermally opened ‘breathing’ end. Finally, hCdc45 binds tightly to longer branched DNA substrates, which would help to stabilize the emerging ‘bubble’ during the initiation of DNA replication and additionally enhance the efficiency of moving of the replicative helicase along DNA (Figure 8B). The displaced strand is guided along Cdc45 in a way that a bulky lesion on this strand does not hinder extrusion (Figure 8D). Because Cdc45 is not an ATP-driven active helicase, power production by the Mcm2-7 complex is likely required to drive the machinery. Therefore, we suggest that Cdc45 is situated in front of the CMG complex and serves as a wedge to separate the two complementary strands where the leading strand is encircled by the Mcm2-7 hexamer and the lagging strand is led along the CMG surface and thereby extruded from the complex (Figure 8D). Although current data are consistent with such a model, alternative and/or additional scenarios are possible. In particular, Cdc45 may mediate stalling and disruption of CMG on replication stress, as proposed recently (55).Because Cdc45 is essential for an active replicative helicase and is used to start origins of replication, it is tempting to speculate that once a bubble has been opened, e.g*.* by a spiral conformation of the Mcm2-7 complex, Cdc45 may act as pathfinder to define the ss/ds junction and keep it open until the spiral form of Mcm2-7 rearranges to the ring-conformation encircling the leading strand.

## SUPPLEMENTARY DATA

Supplementary Data are available at NAR Online.

## FUNDING

The Fritz Lipmann Institute (FLI) is member of the Science Association ‘Gottfried Wilhelm Leibniz’ (WGL) and is financially supported by the Federal Government of Germany and the State of Thuringia. Funding for open access charge: Leibniz Institute for Age Research–Fritz Lipmann Institute.

*Conflict of interest statement*. None declared.

## Supplementary Material

Supplementary Data
