# Supplementary material for: DNA binding properties of human Cdc45 suggest a function as molecular wedge for DNA unwinding
Source: Nucleic Acids Res. 2013 Nov 28;42(4):2308–19. doi: 10.1093/nar/gkt1217 (PMC3936751; doi:10.1093/nar/gkt1217)
Supplement: Supplementary Data [file supp_42_4_2308__index.html]

DNA binding properties of human Cdc45 suggest a function as molecular wedge for DNA unwinding — Supplementary Data 

# DNA binding properties of human Cdc45 suggest a function as molecular wedge for DNA unwinding

## Supplementary Data

files

**Files in this Data Supplement:**

- Supplementary Data - pdf file
- Supplementary Data - docx file
