# Supplementary material for: DNA binding properties of human Cdc45 suggest a function as molecular wedge for DNA unwinding
Source: Nucleic Acids Res. 2013 Nov 28;42(4):2308–19. doi: 10.1093/nar/gkt1217 (PMC3936751; doi:10.1093/nar/gkt1217)
Supplement: Supplementary Data [file supp_gkt1217_SuppFigures1-6_revision_with_removed_red_text.pdf]

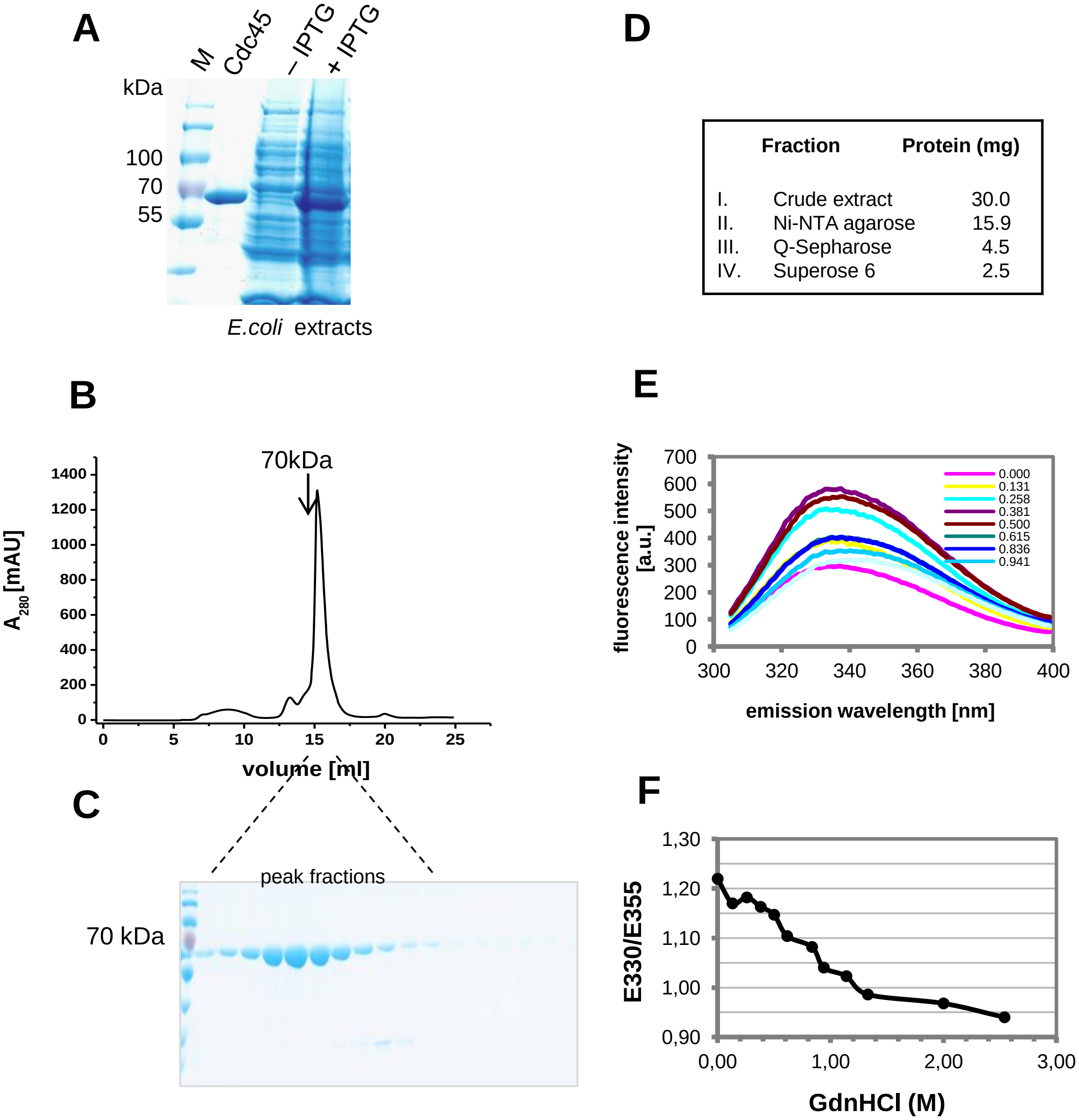

Figure S1. Purification of human Cdc45

C-terminally His_6-tagged Cdc45 was expressed in the *E. coli* Rosetta (DE3) strain under the control of the T7 promoter. Different temperatures, times and durations of gene induction, and different IPTG concentrations were examined to identify conditions under which Cdc45 protein expression was maximal. **A)** Optimal conditions involved treatment with 0.3 mM IPTG, and an overnight induction at 16°C. **B)** Monodisperse of hCdc45 was obtained after purification through Ni-NTA beads, Q-Sepharose and Superose 6. The hCdc45 protein eluted as a narrow peak at a position corresponding to a molecular weight of approximately 65 kDa. **C)** The purity of hCdc45 was examined by SDS-PAGE. **D)** About 2.5 mg of Cdc45 protein were obtained from 2.4 liters of bacterial culture. **E)** Tryptophan fluorescence spectra of hCdc45 in the presence of increasing concentration of guanidinium chloride. F) Denaturation profile of hCdc45 protein. The tryptophan fluorescence was measured after excitation at 295 nm. The maximum of emission of Trp residues in a hydrophobic environment was at 335 nm, whereas in a hydrophilic environment it occurred at 355 nm. A shift from 335 nm to 355 nm indicated the denaturation profile of the protein.

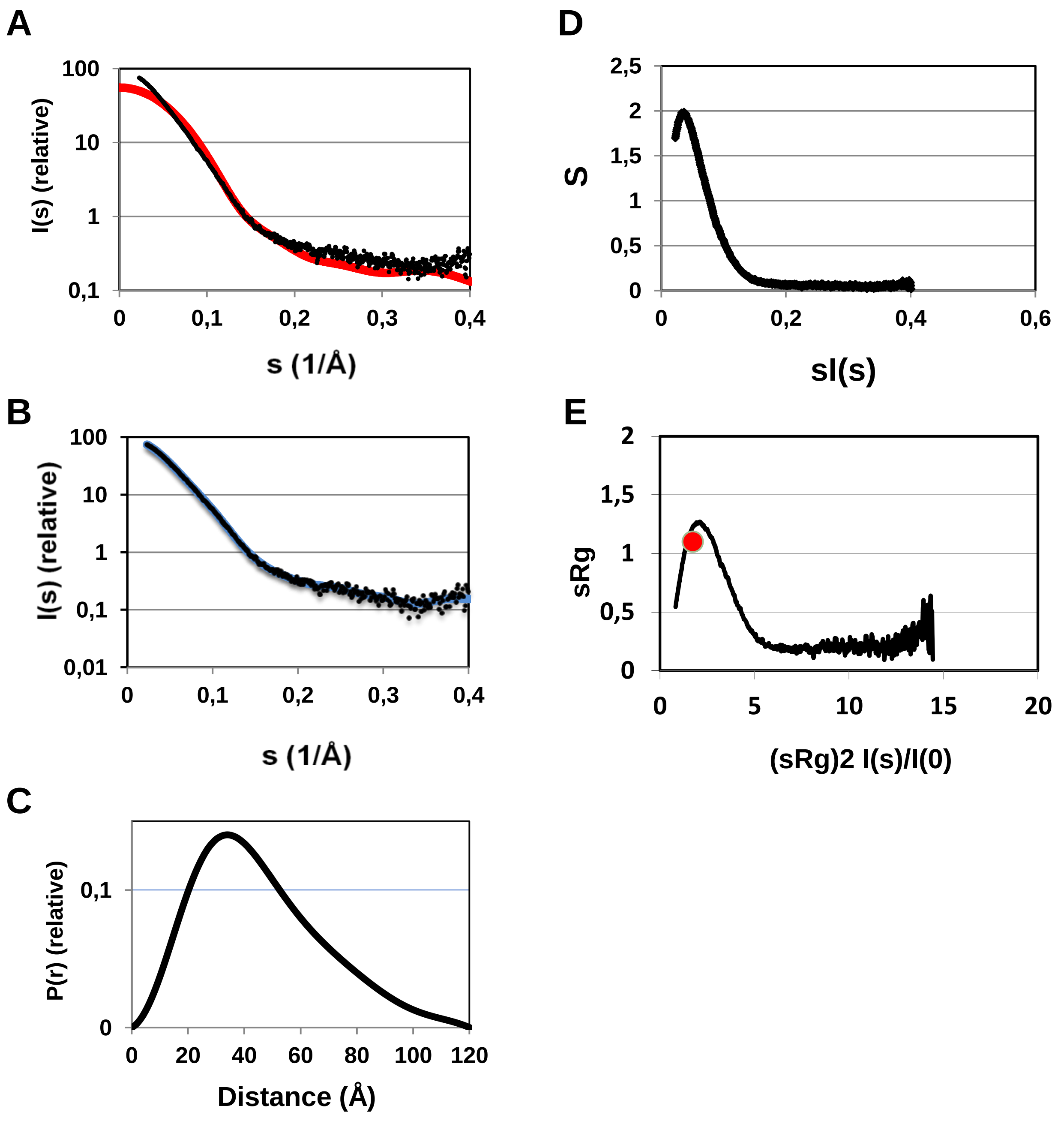

Figure S2. Small angle X-ray scattering

A) The black dots represent the SAXS scattering curve of hCdc45, and the red line the calculated profile of RecJ. **B)** The black dots show the experimentally gained SAXS scattering curve, while the blue line illustrates the calculated curve from GASBOR modeling. **C)** Distance distribution function for hCdc45 as calculated by GNOM. **D)** A plot of s*I(s), which flattens at low s values close to zero, is an indication of a well-folded particle. This plot was further used to determine V_c and the molecular weight without concentration information. **E)** The dimensionless Kratky plot has a strong maximum at S*Rg = 2.0, which suggests a very low amount of disordered structure. The red dot indicates the theoretical position of the maximum for a rigid globular particle at (1.10, sqr(3)).

A

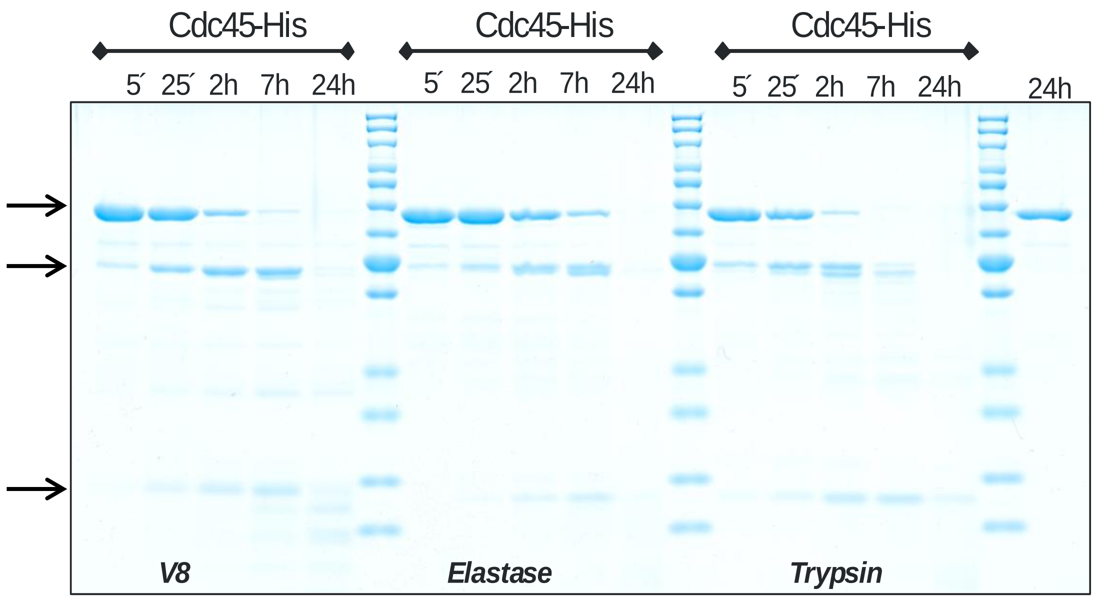

B

```
  1 MFVSDFRKEFYEVVQSQRVLLFVASDVDALCACKILQALF
 41 QCDHVQYTLVPVSGWQELETAFLEHKEQFHYFILINCGAN
 81 VDLLDILQPDEDTIFFVCDTHRPVNVVNVYNDTQIKLLIK
121 QDDDLEVPAYEDIFRDEEEDEEHSGNDSDGSEPSEKRTRL
161 EEEIVEQTMRRRQRREWEARRRDILFDYEQYEYHGTSSAM
201 VMFELAWMLSKDLNDMLWWAIVGLTDQWVQDKITQMKYVT
241 DVGVLQRHVSRHNHRNEDEENTLSVDCTRISFEYDLRLVL
281 YQHWSLHDSLCNTSYTAARFKLWSVHGQKRLQEFLADMGL
321 PLKQVKQKFQAMDISLKENLREMIEQSANKFGMKDMRVQT
361 FSIHFGFKHFLASDVVFATMSLMESPEKDGSGTDHFIQAL
401 DSLSRSNLDKLYHGLELAKKQLRATQQTIASCLCTNLVIS
441 QGPFLYCSLMEGTPDVMLFSRPASLSLLSKHLLKSFVCST
481 KNRRCKLLPLVMAAPLSMEHGTVTVVGIPPETDSSDRKNF
521 FGRAFEKAAESTSSRMLHNHFDLSVIELKAEDRSKFLDAL
561 ISLLSHHHHHH
```

C

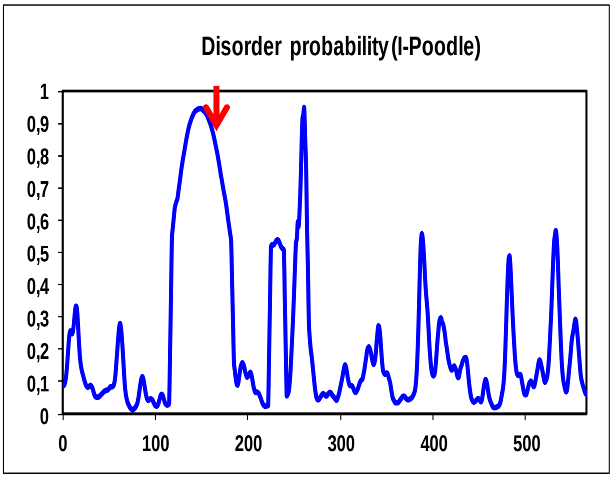

Figure S3. Limited proteolysis of human recombinant Cdc45 protein and bioinformatic prediction of disordered regions using I-Poodle

A) 50 µg Cdc45 were incubated in 100 µl 20 mM Tris-HCl, pH 7.5, and 150 mM NaCl at 25 °C with 1µl of 0.5mg/ml elastase and V8 proteinase and 0.05 mg/ml trypsin, respectively. At the given time points, 10 µl of the reaction mixture was withdrawn and proteolysis was stopped by the addition of 1 µl 100 mM phenylmethanesulfonyl fluoride (PMSF) and 3 µl 4-fold SDS-PAGE loading buffer. Then, the reaction products were analyzed by SDS-PAGE. The bands indicated by arrows were further characterized by Edman sequencing (PRocise 494A, Applied Biosystems). **B)** The amino acid sequence of human Cdc45 protein with region covering the most prominent digestion sites of all three proteases is highlighted in red. V8 cleavage occurred in the glutamate-rich stretch at amino acid 161, elastase cleavage after valine 165, and trypsin cleavage in the arginine-rich region after amino acid 170. **C)** Prediction of disorder probability of human Cdc45 generated by I-Poodle. The red arrow indicates the position of the digestion sensitive region.

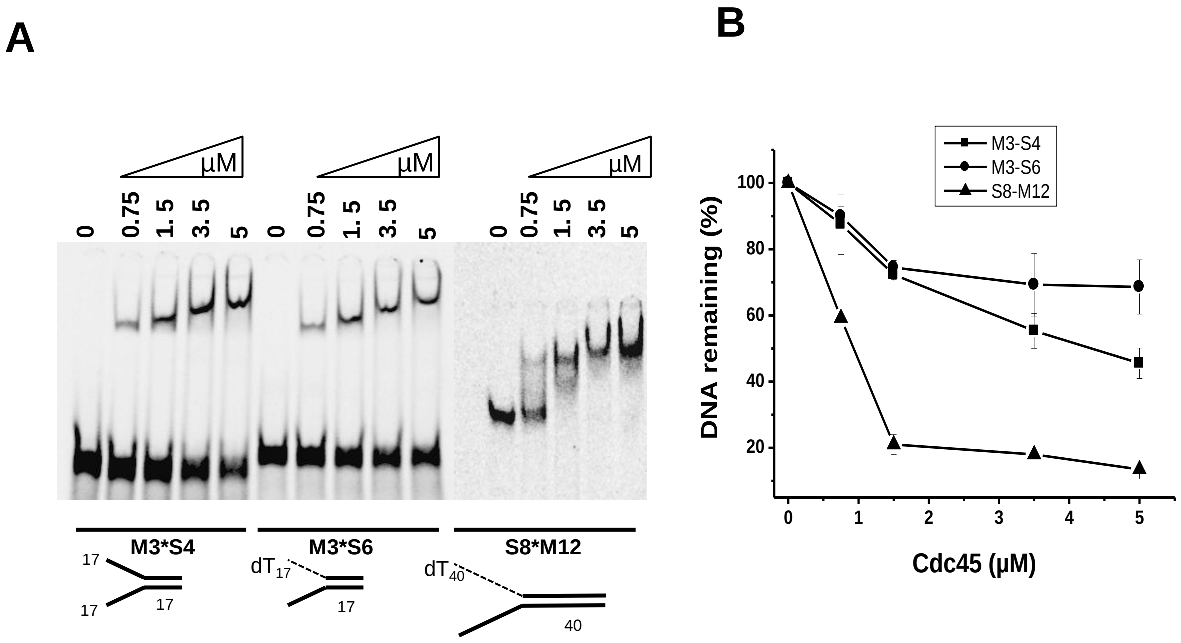

Figure S4. Binding of human Cdc45 to various Y-shaped DNA structures

A) Electrophoretic mobility shift assays were performed using 2 nM 5'-end labeled DNA substrates and the indicated amounts of hCdc45 protein. After 10 min incubation at 30° C, the reactions were stopped by cooling on ice and by addition of 5 µl of loading dye. Samples were separated by electrophoresis through a 10% non-denaturing polyacrylamide gel in TBE buffer. Then, gels were dried at 80°C for 1 h and exposed overnight to a phosphorimaging screen. Gels were visualized using a Phosphor-Imager (Typhoon Trio; GE Healthcare). The synthetic oligonucleotides used are depicted on the bottom of the gel, where the asterisks (*) represent the 5'-end labeled oligonucleotides. **B)** The graph represents the percentage of DNA remaining, i.e. unbound DNA as calculated by using the Image Quant software. Error bars represent the standard deviation of three independent experiments.

A

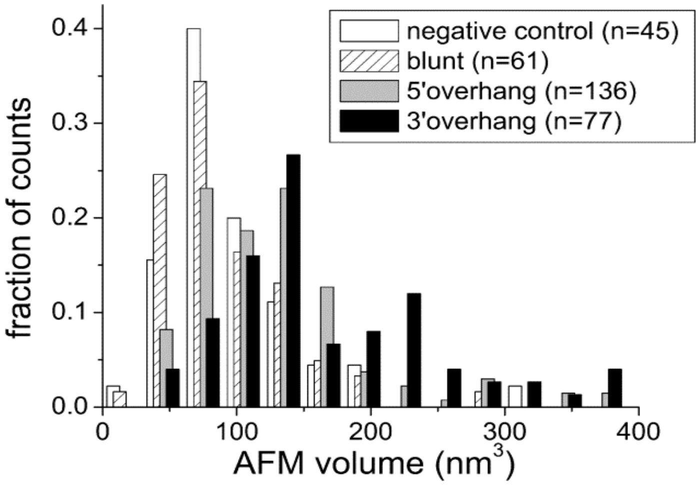

B

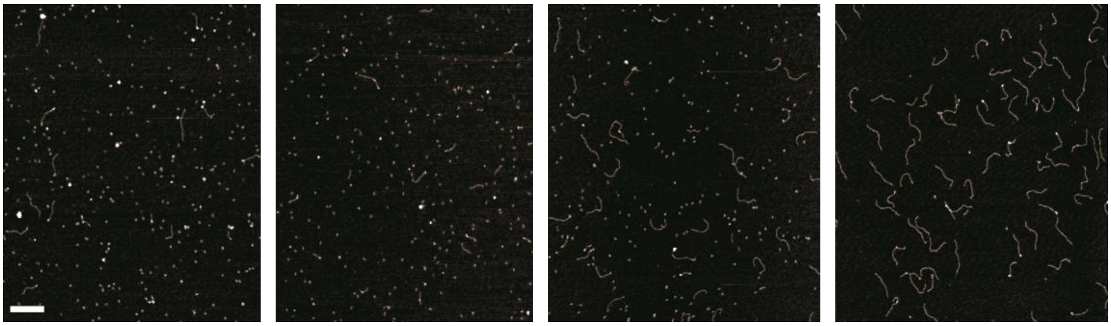

3’ – overhang **5’ – overhang** **blunt DNA** **negative control**

Figure S5. Analysis of Cdc45-binding to 3’ & 5’ overhangs by AFM

A) AFM volume analysis of binding of Cdc45-binding to 3’ overhang (black bars), 5’ overhang (grey bars) and blunt (striped bars) DNA substrates. DNA showed slightly enhanced volumes at fragment ends also in the absence of protein (negative control, white bars), likely due to destabilized DNA attracting microsalt crystals from solution and/or standing up from the mica substrate. The blunt DNA substrates showed similar volumes in the presence of Cdc45, and only a minor species with higher volume (grey arrow, abut 140 nm3 corresponding to 120 kDa). AFM volume analysis revealed higher volume states for 3’ & 5’–overhangs (grey and black arrows, corresponding to around 200 kDa), as determined from Gaussian fits to the data. Since we did not subtract the underlying DNA volume from these peaks (maximum white bars), which corresponds to approximately 70 kDa in this distribution, these peak volumes (grey and black arrows) are consistent with monomers or dimers of Cdc45 bound to the DNA ends, respectively. **B)** AFM images of hCdc45 together with DNA containing 3’ and 5’-overhangs with 40nt tail as well as blunt DNA ends. The scale bar in the left image corresponds to 100 nm, all images sizes are identical.

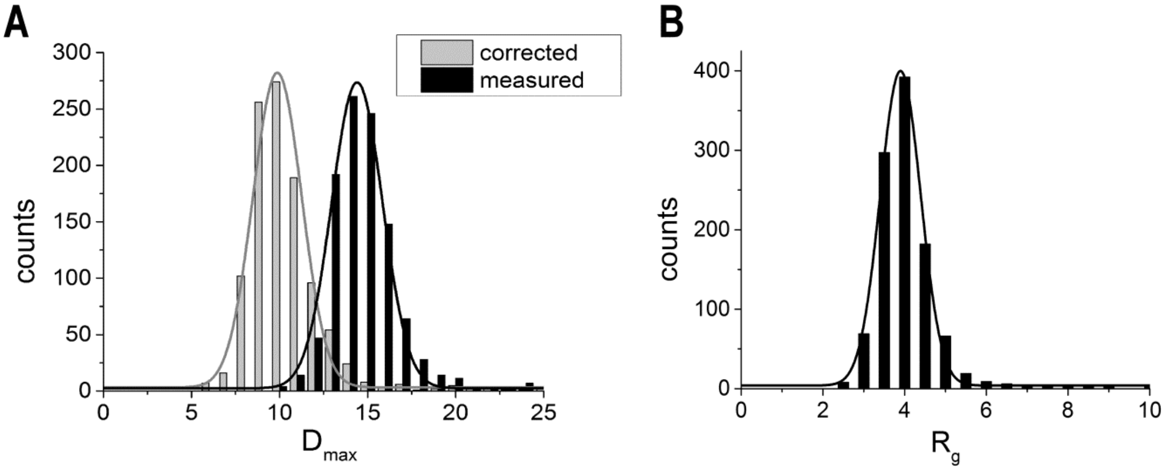

FigureS6. Structural characterization of hCdc45 from AFM measurements.
A) D_{max} = (12.2 ± 3.2) nm, determined as the average from measured and AFM tip convolution corrected particle dimensions in AFM images. In this analysis, dimensions measured directly in the AFM images serve as maximum limits while AFM tip contribution corrected dimensions provide the minimum limits of the range of occurring D_{max} values. Only particles with molecular weights in the range of 63 kDa ± 2 standard deviations (SD; see Figure 1B) were included in the analyses. Uncorrected D_{max} values ("measured") were obtained as the lengths of the long axes of an ellipse describing the particles in the AFM images. These lengths were corrected for contributions from the finite dimensions of the AFM imaging tip ("corrected" D_{max}) using the broadening of DNA widths in the images compared to their theoretical widths of ~ 2 nm (the diameter of the DNA double helix) as a calibration standard, as previously described (55). Measured and AFM tip effect corrected D_{max} values for hCdc45 were obtained as the centers of Gaussian fits (black and grey lines, respectively, with $R^2 > 0.98$) to the distributions of individual D_{max} values (n = 1047 particles from 3 experiments using 4 different AFM tips). **B)** R_g = (3.9 ± 0.5) nm, determined as the center of a Gaussian fit to particle radii (n = 1066, fit quality $R^2 > 0.99$). Particle radii were obtained as the half averages of major and minor elliptical axes, corrected for AFM tip contributions. Good reproducibility was apparent from small variations between separate experiments resulting in comparable D_{max} and R_g values obtained as the averages from the individual experiments with errors calculated using error propagation (D_{max} = (12.6 ± 2.6) nm; R_g = (4.3 ± 0.7) nm).
